# Towards Digital-Twin Assisted Software-Defined Quantum Satellite Networks

**DOI:** 10.3390/s25030889

**Published:** 2025-01-31

**Authors:** Francesco Chiti, Tommaso Pecorella, Roberto Picchi, Laura Pierucci

**Affiliations:** Department of Information Engineering, University of Florence, Via di Santa Marta 3, 50139 Firenze, Italy; francesco.chiti@unifi.it (F.C.); tommaso.pecorella@unifi.it (T.P.); laura.pierucci@unifi.it (L.P.)

**Keywords:** quantum digital twin, quantum internet, quantum key distribution, quantum satellite backbone, quantum software-defined networking

## Abstract

The Quantum Internet (QI) necessitates a complete revision of the classical protocol stack and the technologies used, whereas its operating principles depend on the physical laws governing quantum mechanics. Recent experiments demonstrate that Optical Fibers (OFs) allow connections only in urban areas. Therefore, a novel Quantum Satellite Backbone (QSB) composed of a considerable number of Quantum Satellite Repeaters (QSRs) deployed in Low Earth Orbit (LEO) would allow for the overcoming of typical OFs’ attenuation problems. Nevertheless, the dynamic nature of the scenario represents a challenge for novel satellite networks, making their design and management complicated. Therefore, we have designed an ad hoc QSB considering the interaction between Digital Twin (DT) and Software-Defined Networking (SDN). In addition to defining the system architecture, we present a DT monitoring protocol that allows efficient status recovery for the creation of multiple End-to-End (E2E) entanglement states. Moreover, we have evaluated the system performance by assessing the path monitoring and configuration time, the time required to establish the E2E entanglement, and the fidelity between a couple of Ground Stations (GSs) interconnected through the QSB, also conducting a deep analysis of the created temporal paths.

## 1. Introduction

Innovative Quantum Networks (QNs) are conceived with the aim to interconnect multiple Quantum Computers (QCs). The interconnection between multiple QNs will lead, as was the case for the classical Internet, to the creation of a global QN: the so-called Quantum Internet (QI) [1]. In QNs, the created quantum states can be teleported to arbitrarily long distances [2] through the quantum teleportation procedure. In order to perform these operations, pairs of entangled particles are exchanged throughout quantum channels, and classical communication channels are established through the same connections to enable the optimal configuration of the network and transport the information about the quantum teleportation measurements [3,4,5,6,7,8].

This network will enable previously unavailable functionalities and also allow for the interconnection of computers with a high level of security. Furthermore, through QI, remote QCs can be interconnected in order to communicate and cooperate with the aim of solving extremely complicated computational tasks operating as a distributed quantum computing system. In fact, the obtained system constitutes a single Quantum Device (QD), which is characterized by a number of qubits that scales linearly with the involved remote QCs and whose computational capacity increases exponentially [1,9,10,11].

However, despite the recent acceleration in the development of quantum technologies, the generation rate of high-fidelity quantum states decreases exponentially with distance due to Optical Fiber (OF) losses [5]. Therefore, performing a large-scale entanglement distribution is still an open issue. To mitigate the transmission problems, it is necessary to introduce specific devices called Quantum Repeaters (QRs). QRs are dedicated devices equipped with Quantum Memories (QMs) that allow for the storage of intermediate quantum states [12]. The whole communication channel is divided by the QRs into several links and the creation of entanglement between neighboring QRs is feasible through the transmission of photons entangled with their QMs. The entanglement swapping procedure is performed between multiple adjacent nodes using specific schemes allowing the generation of the entanglement on an End-to-End (E2E) basis. Specifically, the entangled pairs *a* and *b* and *c* and *d* are processed in order to obtain a new Einstein–Podolsky–Rosen (EPR) pair between *a* and *d*, which are not related through entanglement, by conducting a Bell State Measurement (BSM) on particles *b* and *c*.

In view of the fact that photons usually experience negligible losses in space vacuum, free-space satellite links have recently been considered as a possible solution for the problems related to OFs, making the transmission of photons over a continental scale feasible. Moreover, QMs and other QR-related devices such as dilution refrigerators have already been tested in the space environment [13,14], and inter-satellite optical links have been successfully demonstrated in ESA missions [13]. Therefore, the realization of so-called Quantum Satellite Repeaters (QSRs) can be considered feasible [15,16]. Specifically, as explained in [13,14], different types of quantum memories exhibit varying storage times. While some have very short durations, others can retain quantum states for up to tens of seconds under specific conditions. Equipping satellites with these advanced memories could allow the storage of entangled pairs until entanglement swapping operations are completed over global-scale E2E satellite communication paths.

As reported in [17], LEO satellites, unlike Medium Earth Orbit (MEO) or Geostationary Earth Orbit (GEO) satellites, face unique challenges. These include limited coverage and shorter lifespans due to atmospheric drag. Achieving global coverage with LEO satellites requires the deployment of large constellations, which increases management complexity. However, the issue of mobility and its management can be effectively addressed with SDN and specific algorithms operating on Temporal Graphs (TGs) [18]. Moreover, as the elevation of the satellite relative to the Ground Station (GS) decreases, the atmospheric path to be traversed may increase significantly, thereby amplifying the impact of scattering on the entangled state. However, LEO satellites offer distinct advantages for quantum communications. Attenuation and latency are also lower for inter-satellite links due to their shorter length compared to links between MEO or even GEO satellites. Additionally, shorter distances enhance the probability of successful entanglement generation and increase entanglement rates [19]. Furthermore, additional studies are required to understand the impact of issues associated with the upper layers of the atmosphere.

Nevertheless, the creation of a QN on a global scale necessitates the conceptualization of dedicated architectures and protocols, which must also consider the integration of different technologies while ensuring their interoperability [20]. In the case of a classical distributed computing system, several services are located on multiple computers; therefore, the system response time is significantly conditioned by the communication over the network. This condition is also evident in distributed quantum systems, where the diverse higher-level operations dictated by the quantum algorithm require proper allocation among the memories of the involved QCs. Moreover, QMs currently have significantly limited storage time; therefore, the design and implementation of completely distributed QN management protocols are particularly difficult [21,22]. Software-Defined Networking (SDN) is considered a promising architecture to overcome the limitations of a fully distributed algorithm. Indeed, in a QN, the SDN Controller can manage the Bell pair distribution mechanisms on an E2E basis.

**Figure 1:**
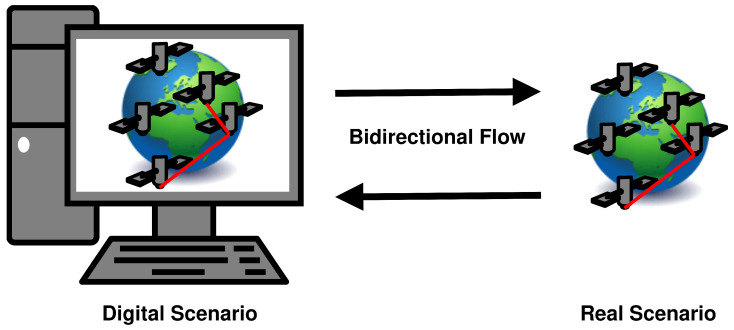
Representation of a DT data flow.

The management of such a complex system needs to be accurate, and a Digital Twin (DT) of a Quantum Satellite Backbone (QSB) can be a promising solution. As a matter of fact, a DT is a digital thread that synchronizes with physical systems to optimize products or performance. As reported in [23], Digital Model (DM), Digital Shadow (DS), and DT are terms often used as synonyms. Nevertheless, their definitions differ considering that DM, DS, and DT have different levels of data integration between the digital and the physical entities. For DTs, a bidirectional data flow is provided between a physical system and its digital replication, as represented in Figure 1. Therefore, the digital entity is also a controlling instance of the physical object, and a modification in the state of the physical system is directly reflected on the digital replica and vice versa. As reported in [24], a DT can improve the real-time analysis, perform prediction, and improve the control capabilities of SDN to satisfy the requirements of the application scenario. In the case of satellite networks, this means that there exists the possibility to uplink data or commands provided by the DT to improve performance [25]. Specifically, for a QSB, it is necessary to create a Quantum Digital Twin (QDT) [26], which allows for the addressing of potential problems. For instance, upon the detection of an error, the physical network will transmit an error report to the DT, which will then refine the routing schemes and the latest satellite network status using specific algorithms. The improved routing schemes will then be verified in the virtual networks, in which the QSRs chain with the best performance will be calculated and subsequently established in the physical network [27].

Given the above considerations, this paper proposes a QSB composed of Low Earth Orbit (LEO) satellites that can be controlled via a specifically designed Control Plane (CP) studied according to the SDN paradigm that operates in a coordinated manner with a DT. Each element composing the Data Plane (DP) is a QSR, which performs the BSMs and the swapping operations to establish an E2E entanglement between two GSs. Considering that QSBs need to be very accurately controlled, the SDN is recognized to be particularly appropriate to handle these kinds of architectures. In fact, the achievement of high rates requires efficient entanglement generation and swapping operations, a goal that can be easily achieved with the use of SDN [16]. In addition, on the basis that in specific cases, including distributed computing, the E2E operations should be minimized to mitigate the impact of decoherence that induces a deterioration of the entangled quantum states and in order to minimize the overhead that is due to the swapping operations, the paper also proposes a dedicated Network Layer protocol. Specifically, the proposed protocol is utilized to monitor and manage the chain of QSRs according to the pattern given by the solutions processed by the DT. Moreover, the paper defines an optimization procedure to minimize the Round-Trip Time (RTT) of the quantum resource monitoring flows between the DT and the quantum satellites. Therefore, the Master Control Station (MCS) [28] interacts with a DT, which is a digital replica of the entire Quantum Satellite Network (QSN) and the involved QDs on the ground and operates on it through optimization procedures to generate optimal paths and achieve certain performance. The E2E paths are then configured by the SDN Controller integrated in the MCS based on the obtained results. Specifically, the controller performs the setup of the inter-satellite optimized path and coordinates the entanglement generation and swapping procedures between the QSRs and the GSs that compose the E2E path.

In summary, this paper proposes an approach for the management of a QSB network that consists of multiple QSRs with an emphasis on the CP driven by the DT, with the contributions outlined below:

- The design of a DT-assisted SDN-based CP including a specific TN [29,30,31] approach;
- A specifically designed Network Layer optimized protocol to monitor the QSN status and manage the generation of multiple entanglements on an E2E basis;
- A performance evaluation in terms of attenuation, E2E path configuration time, entanglement generation time, the RTT required to reach each satellite, and fidelity.

The manuscript is composed of the following Sections: Section 2 reports a state-of-the-art analysis, focusing on the related scenario and experiments. Section 3 describes the system model and the proposed protocol. Next, Section 5 presents the validation of the results. Finally, Section 6 concludes the manuscript and outlines future developments.

## 2. Related Works

Recently, quantum communications have had remarkable development, and the following step consists of the creation of a network of QCs on a global scale that might allow computations that can be beyond the capacity of a single extremely high-performing QC. The developments in satellite and QMs make this step more feasible [32], and many experiments have already been conducted.

A comparison between the properties of the current satellite-based QNs with those of the OFs has been made in [33]. Moreover, ref. [34] provided a detailed analysis of multiple disturbance effects that the quantum communication channels are subjected to. A specific model that includes the losses typical of the quantum signals during the propagation along the atmosphere is provided in [35].

The LEO Micius satellite has been used to distribute quantum keys between several locations deployed on an intercontinental scale scenario [36,37]. Moreover, the involved research groups conducted several teleportation experiments between the GSs and the satellite [38]. In addition, in [39], an Italian research group has verified the applicability of many quantum cryptography protocols from a satellite to a GS considering four different polarization states for the sent qubits.

Free Space Optics (FSO) technology has been applied in several studies that also involve the area of quantum communications. In particular, it has already been used to perform quantum communication on Earth between the sites of La Palma and Tenerife, directly connecting a GS with another, separated by a distance of 143 km [40]. Furthermore, as reported in [41], FSO has also been used to connect two satellites through an inter-satellite link. Nevertheless, even though the beam wandering and the atmospheric problems limit the performance of FSO, especially in the case of G2S and Satellite-to-Ground (S2G) links, adequate performance can be achieved in favorable meteorological conditions with the 1550 nm wavelength [42].

In [43], several Quantum Key Distribution (QKD) schemes, which utilize entanglement as a resource, were compared on a simple QN composed of three satellites. Moreover, in [44], a multiple-layer architecture of QSNs has been proposed, and a solution that involves GEO and LEO satellites with a specific solution, which uses an algorithm dedicated to the E2E key distribution. Numerous configurations of satellites have been studied with the aim of balancing the total number of involved satellites and the dissemination of the entangled states trying to maximize the entanglement rate [45].

The SDN paradigm is widely employed for the control of traditional satellite networks, including interplanetary networks [46]. However, it is gradually finding application in the control of QSNs as well. In [22], SDN was considered to be an indispensable instrument allowing easy control of the future QNs. Many of the experiments conducted so far have concerned couples of G2S links towards single satellites and require the realization of a dedicated Data Link Layer protocol such as that defined in [47]; however, in order to deploy a constellation, an effective Network Layer protocol should also be designed. An architecture that integrates a single SDN Controller on Earth managing an entire constellation composed of multiple QSRs has been introduced in [20]. Specifically, the study analyzes the performance of several path selection algorithms. Furthermore, ref. [16] examined the performance of some constellations composed of QSRs. Many studies also consider the interaction between multiple domains. Specifically, in [21], an architecture with a modular two-tier CP in which some controllers are deployed in the constellation is proposed. Moreover, a similar approach is described in [48], in which different entanglement generation strategies are also studied.

In [49] several types of architectures that integrate the DT for network management are highlighted. Specifically, the authors state that 68% of the architectures proposed in the literature follow a centralized approach, which involves a unique DT updated with data from the entire network. For instance, ref. [50] proposed a centralized solution based on graph neural networks aimed at optimizing a classical network. In particular, the proposed approach considers the coupling of a DT with an optimizer running on an SDN Controller. Furthermore, in [51] an SDN-DT architecture for the Industrial Internet of Things (IIoT) was proposed. In this case, the network was integrated with the industrial components that compose the system. The authors argued that the SDN controller collects all data describing the physical network, builds the network topology model, and supplies the essential intelligence to the DT. Moreover, the Third-Generation Partnership Project (3GPP) produced a technical report on the management aspects of Network Digital Twins (NDTs) [52].

Recently, technologies such as DTs have also found applications in the quantum communications field. For instance, DT technologies applied to the IIoT were explored in [53]. Specifically, the paper proposed a channel encryption scheme based on quantum communications to guarantee communication security. In [54], the components of a possible Spacecraft Digital Twin (SDT) were discussed. In particular, the paper described the structure, including the data source, the system configuration, and the data service mode. Furthermore, ref. [25] analyzed the utility of the DT in both the design phase of a satellite network and for tuning and synchronization of the deployed physical systems. In [27] an Intelligent DT-based Software-Defined Vehicular Network (IDT-SDVN) scheme was proposed. Specifically, the IDT was constructed in the controller to perform a predictive verification before the application of a generated functional model as well as the maintenance diagnosis of operative networks in real scenarios.

Few studies have been pursued so far on specific DT-based architectures for QSNs. Moreover, the application of a specific networking protocol and the theory of TNs on QNs composed of LEO satellites is a research area that should be further explored. Therefore, this manuscript, in addition to defining a possible architecture, proposes a networking approach based on the TN theory and a specific protocol for the E2E entanglement generation between a couple of GSs positioned at the antipodes by employing multiple QSRs.

## 3. System Model

**Figure 2:**
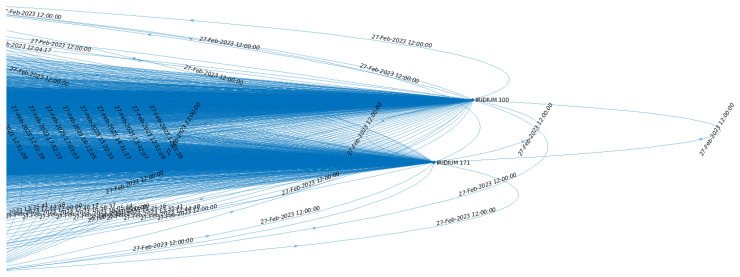
A detail of the TG of the constellation. Specifically, the instants of time from which the links stop being available are represented.

A QSB can ensure significantly better performance than OFs. However, an efficient QSB requires extremely precise control, and a DT working in synergy with an SDN Controller is considered essential to achieving this objective. A DT designed to manage a quantum constellation effectively must include a reliable model of the entire network, the links, and the main parameters that characterize the involved devices. In the case of a LEO satellite network, where the scenario evolves rapidly due to the high velocity of the satellites, a TG can constitute an excellent instrument for appropriately representing the scenario evolution. Moreover, an efficient temporal path selection algorithm operating on a TG can be a solution to enhance the entanglement rate and fidelity on the designated path. Specifically, a TG can be expressed as G=(V,E), with V and E, which are, respectively, the sets of vertices and edges that compose G [29]. Every single edge e∈E consists of the following parameters $\left(u,v,t_{start},t_{end},W_{u,v}\right)$. In particular, u,v∈V are the two adjacent nodes, and $t_{start}$ and $t_{end}$ are the *start time* and the *end time*, the latter depicted in Figure 2. In the case of satellite networks, a TG can be derived from the Two-line Element Sets (TLEs) [55], a specific data format that encodes a list of satellites that compose a constellation.

In this paper, we propose to apply a method for optimizing the flows between the DT and the satellites that compose the E2E paths. The optimization strategy was derived from that reported in [56] and is expressed as follows:

(1) $$\begin{array}{cc} \min_{f_{uv}}\max_{k\in D}\; & {\mathrm{RTT}}_{k}=\;\;\;\;\sum_{\left(u,v\right)\in P_{k}}\frac{d_{u,v}}{c}\;+\;\;\;\;\sum_{\left(v,u\right)\in P_{k}}\frac{d_{v,u}}{c},\;\forall k\in D\\ s.t.\; & \sum_{v\in N(u)}f_{u,v}-\;\;\;\sum_{v\in N(u)}f_{v,u}=\left\{\begin{array}{cc} r_{k}-f_{s,k}\approx r_{k} & \mathrm{if}\;u=k,\;k\in D\\ \sum_{k\in D}f_{s,k}-\sum_{k\in D}r_{k}\approx -\sum_{k\in D}r_{k} & \mathrm{if}\;u=s\\ 0 & \mathrm{otherwise} \end{array}\right.\\ \; & 0\leq f_{uv}\leq C_{u,v},\;\forall \left(u,v\right)\in E\\ \; & f_{uv}=0,\;\forall \left(u,v\right)\notin E \end{array}$$

where the parameters and the related constraints are defined as follows:

- *s*: DT node.
- D⊆V: Set of destination nodes.
- $f_{u,v}$: Flow of data on edge (u,v).
- $d_{u,v}$: Distance on edge (u,v).
- *c*: Speed of light.
- $C_{u,v}$: Capacity of edge (u,v).
- $r_{k}$: Data rate at destination node k∈D.
- $P_{k}$: Path from the DT to satellite *k*.
- N(u): Set of neighbors of node *u* in the network.
- ${\mathrm{RTT}}_{k}$: RTT from the DT to satellite *k*.

Specifically, the optimization problem in Equation (1) minimizes the maximum RTT between the DT and each satellite, aiming to reduce the RTT for the slowest one. In the proposed architecture, the DT node, denoted as node *s*, functions as a client in a client–server model that performs a request and aggregates responses from multiple servers. This characteristic is reflected by considering the incoming flow, that is, the overall flow of response data to be significantly greater than the outgoing flow, i.e., the data flow of the requests. As a result, the expression for *u* equal to *s* is defined as negative. Conversely, in the case where *u* is equal to *k*, the result is equivalent to $r_{k}$, as the flow from the DT to node *k* is assumed to be negligible.

One of the parameters that a DT node must consider is the attenuation between the links that compose the paths. As reported in [57,58], the attenuation between the GS and the satellite in contact is modeled as follows:

(2) $$A_{u,v}\left(t\right)=\frac{Z_{u,v}^{2}\left(t\right)\left(\Theta_{T}^{2}\left(t\right)+\Theta_{med}^{2}\left(t\right)\right)}{D_{R}^{2}T_{T}\left(1-L_{P}\right)T_{R}T_{OPT}PDE}10^{\frac{A_{med}\left(t\right)}{10}}$$

where $Z_{u,v}\left(t\right)$ is the distance between the GS and the satellite telescopes or between two telescopes belonging to two neighboring satellites and $\Theta_{T}\left(t\right)$ and $\Theta_{med}\left(t\right)$ are, respectively, the divergence angle of the transmitting telescope and the divergence angle caused by the atmospheric turbulence, expressed as follows:

(3) $$\Theta_{T}\left(t\right)=1.27\frac{\lambda }{D_{T}}$$

(4) $$\Theta_{med}\left(t\right)=2.1\frac{\lambda }{r_{0}}$$

Moreover, $D_{T}$ and $D_{R}$ are the diameters of the transmitter and receiver telescopes apertures, λ is the wavelength, $T_{T}$ and $T_{R}$ are the transmission factors of the transmitter and receiver telescopes, $T_{OPT}$ models the optics between the entangled pair sources and the satellite’s telescopes, $L_{P}$ is the pointing loss due to their misalignment, PDE is the single photon detector efficiency, and $A_{med}$ is the medium attenuation, calculated as in [12]. Considering that LEO constellations operate above the Karman line [59], which is the conventional definition of the edge of space, for the inter-satellite links, the value of $A_{med}$ was considered to be zero. Therefore, in this case, the geometric component prevails in Equation (2).

Another parameter typically measured in QNs which has been analyzed in Section 5 is fidelity. As explained in [2], the fidelity between two quantum states described by the density matrices ρ and σ is given by the following formula:

(5) $$F\left(\rho ,\sigma \right)={\left(\mathrm{Tr}\sqrt{\sqrt{\rho }\sigma \sqrt{\rho }}\right)}^{2}$$

This formula is used to measure how close two quantum states are to each other, with values ranging from 0 to 1, where 1 indicates identical states. The E2E flows between the two GSs can be optimized by the DT through the application of the optimization method defined in [18], also taking into account parameters such as fidelity among the problem constraints.

Furthermore, since the management of the swapping process can cause an increase in terms of overhead, it is appropriate to minimize it, especially in the case of distributed computing operations. This target can be achieved with an effective control of entanglement generation and swapping procedures by the CP, an objective that can be achieved through the optimization problem defined in [18].

## 4. Proposed Architecture

As reported in RFC 9340 [3], there are significant differences between classical networks and QNs concerning data operation, network resource management, and the physical principles governing the quantum world. For instance, differently from classical networks that operate according to the “store and forward” mechanism, QNs operate following the “store and swap” logic that requires different state management techniques. Moreover, the control algorithms and QNs optimization procedures are different from those for classical networks, in the sense that entanglement swapping is stateful in contrast to stateless packet-by-packet forwarding. Therefore, it is necessary to define specific architectures and protocols for the proper management of QNs resources.

As mentioned in the previous sections, a LEO satellite constellation where each satellite is a QSR is a promising solution for long-distance quantum communications. Therefore, we consider a LEO constellation where all elements that compose the network are QSRs. The QSRs and the GSs are part of the DP, and work in the FSO frequency range [60]. The performance of FSO is significantly conditioned by atmospheric factors that cause phenomena such as beam wandering, especially regarding G2S and S2G communications [35]; however, acceptable performance can be achieved in favorable meteorological conditions with the use of the 1550 nm technology.

**Figure 3:**
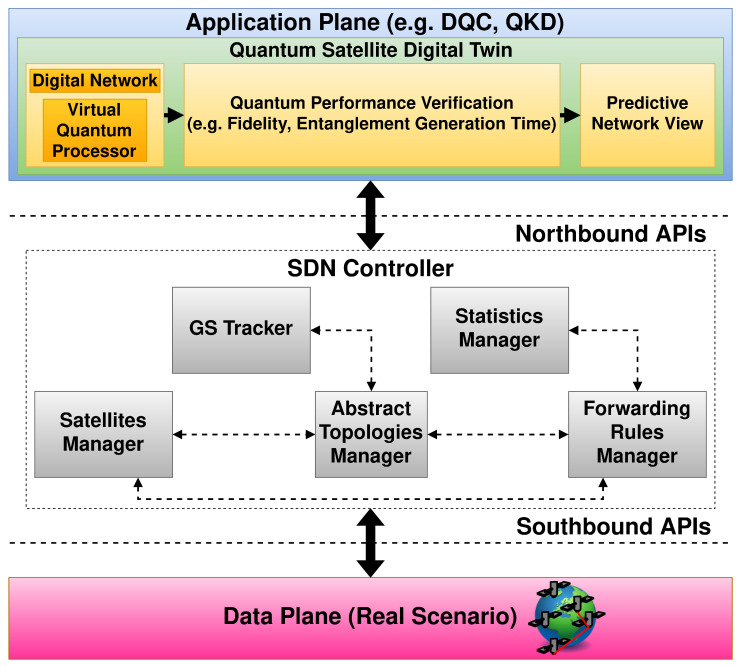
The proposed QSB control architecture. The DT monitors the network and calculates the best E2E temporal paths. Moreover, the SDN Controller configures the satellite paths resulting from the processing performed by the DT.

The main problems in managing such a system are related to calculation of the best path according to the satellite positions and available resources, which vary over time. Our proposed architecture, shown in Figure 3 is composed of the satellite LEO QSRs network, a DT, and an SDN Controller:

- The DT performs the network optimization and the what-if analysis to find the optimal path in the QSRs network.
- The SDN Controller is in charge of applying the configurations to the QSRs, which acts as SDN switches.

Specifically, we have defined the modules composing the Quantum Satellite DT (QSDT) as follows:

- A first block containing the digital network and a global vision of the involved quantum processors, i.e., the virtual quantum processor.
- A second block that performs an evaluation of the QN’s performance, also accounting for potential faults.
- A third block that includes the network view derived from the performed simulations.

Furthermore, the modules that constitute the SDN Controller and have a direct interaction with the QSDT are outlined below:

- The Abstract Topologies Manager is tasked with the storage and management of information concerning the networking devices under its supervision. It observes notifications and updates the topology data, specifically the temporal graph, which includes all satellites forming the constellation along with their connections.
- The Satellite Manager provides information on the satellites and their ports, allowing Northbound APIs to retrieve details about the constellation.
- The Forwarding Rules Manager (FRM) handles basic forwarding rules, resolving conflicts and validating them. Specifically, given that the TN module offers a temporal perspective of the scenario, the FRM can adjust satellite forwarding rules based on the scenario’s temporal progression.
- The Statistics Manager collects statistics by sending requests to all active satellites and storing the responses within the operational statistics subtree.
- The GS Tracker retains data on the GSs and offers APIs to access information about end nodes.

The DT derives a virtual scenario extrapolating a TG from the TLE data. The TG includes the time intervals at which the satellites are in contact with each other, and the average inter distances as weights. Afterward, a specifically designed path selection algorithm used to calculate the best path considering the time intervals in which the path is viable is applied to the TG and performance is verified to meet the user’s requirements.

The SDN Controller provides the configuration of the satellite path, handling entanglement generation and swapping in an appropriate manner, communicating with the satellites via dedicated southbound APIs.

### Quantum Path Setup Protocol

In the following part, we describe the protocol that has been designed for the architecture shown in Figure 3, along with its packet format.

Specifically, the packet design is based on the TCP/IP stack and uses the format defined in RFC 5444 [61], which is related to the Mobile Ad Hoc Network (MANET) Packet/Message Format, but it can be easily extended. The packet is composed of the following fields:

- Type: this is a field used for defining the type of the message. Specifically, the four message types are defined as follows:
  – Type 0: sent by a GS to the MCS indicates a request to initiate a quantum communication with a specific destination.
  – Type 1: sent by the MCS to a destination GS to verify whether it is willing to establish a quantum communication.
  – Type 2: sent by the MCS to the source GS to confirm that the destination GS is available to initiate a quantum communication.
  – Type 3: sent by the MCS to the QSRs to perform the communication setup according to the specifications provided by the DT.
  – Type 4: sent by the DT to the MCS to communicate the list of satellites to be involved in quantum communication. The list is obtained from the optimization performed by the DT.
  – Type 5: sent by the satellites to the DT to communicate the status of their quantum memories.
- T_start_ + Duration: specifies the initial time and duration of the path that has to be initialized. It allows programming of the establishment and termination of the connections at specific times.
- Flow descriptor: variable length field, dependent on the protocol stack being used.

The above field description the “Flow descriptor” field depends on the protocol stack being used.

The time and duration fields need to be further specified, since that they use a standardized time representation. This point is subject to further investigation, and it shall be considered in the implementation and standardization phases. In our work we did use the standard UNIX time representation.

The DT processes the connection requests originated by one of the GSs. Specifically, the DT receives a message with Type equal to 0 and the flow identifier, e.g., the IP address of the GS that requests the connection, the IP address of the destination GS, etc. Moreover, the DT sends a Type 1 message to the other GS to verify if it can accept the connection. If the destination GS acknowledges the request, the DT forward the Type 2 response message to the GS that had requested to communicate to confirm that the receiving GS is available. The DT establishes a connection with the involved satellites in order to obtain information regarding the quantum memory status. The satellites transmit Type 5 messages to the DT, enabling it to update the state of its digital network. Therefore, the DT starts to compute the best E2E paths for all the time intervals wherein the GSs are simultaneously in contact with the favorable satellites using the proposed optimization procedure, which minimize the RTT between the DT and the involved satellites and derives a list of available paths.

The list is sent to the controller installed in the MCS with a Type 4 message. The derived time intervals will form the Duration field of the subsequent messages explained in the following part. Specifically, the Controller sends multiple packets to the QSRs, with Type set to 3. In this case, the Duration contains the time interval in which the path is valid, and it has to be considered by the node in order to operate during the specified time interval. Furthermore, the source port and the destination port define the quantum flows, which are mapped in specific quantum memory locations on the QSRs that compose the chain.

**Figure 4:**
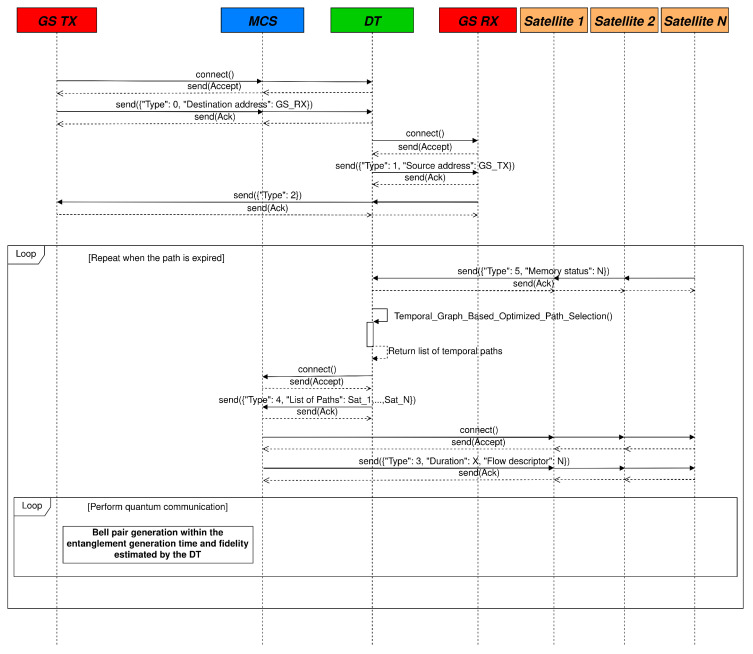
Sequence diagram of the proposed protocol.

Therefore, quantum communication can begin and concludes when the path ceases to exist. At that point, the procedure restarts in order to establish a new E2E path for the following time interval. A sequence diagram of the protocol is represented in Figure 4.

## 5. Results

The developed framework with which we conducted the simulation campaign described in this Section was run on a machine with an Intel Core i7-8750 CPU at 3.2 GHz with 16384 MB of RAM and Ubuntu 22.04.5 LTS installed. Specifically, the software was developed using the satellite toolbox of Matlab [62] together with Netsquid [63], a Python library for the development of QNs. The framework interprets the data of the TLEs available in [64] to create the TG of the satellite constellation, which is composed of 75 satellites.

The simulation has been conducted during a time interval of 6 h, creating a TG with an accuracy of 1 s for the time intervals. The two GSs were positioned at a distance of 20,000 km one from the other, placed on the Equator at the geographic coordinates (0°, 0°) and (0°, 180°). Considering that, as reported in [65,66], the upper limit of the troposphere can reach 20 km, the section of the G2S links affected by atmospheric conditions was calculated considering this altitude. Moreover, given that the creation of a G2S or S2G quantum link is practically impossible under suboptimal meteorological conditions [67], we have considered an atmospheric attenuation of 0.032 $\frac{\mathrm{dB}}{\mathrm{km}}$, which corresponds to a visibility of 100 m for a wavelength of 1550 nm, as reported in [12]. The parameters and the respective values adopted in the simulations to model the optical communication apparatuses are shown in Table 1.

**Figure 5:**
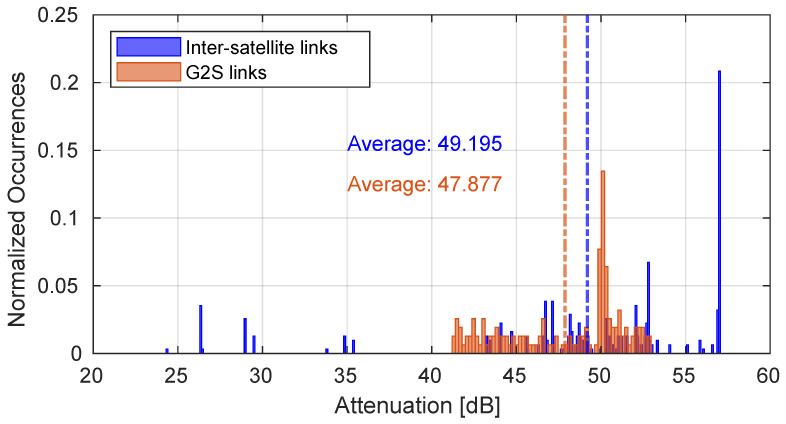
Attenuation on the considered G2S and inter-satellite links.

**Figure 6:**
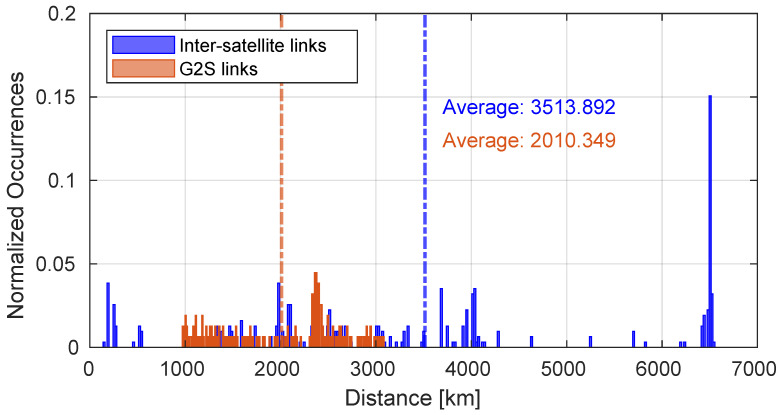
Length of the links that characterize satellite paths.

**Figure 7:**
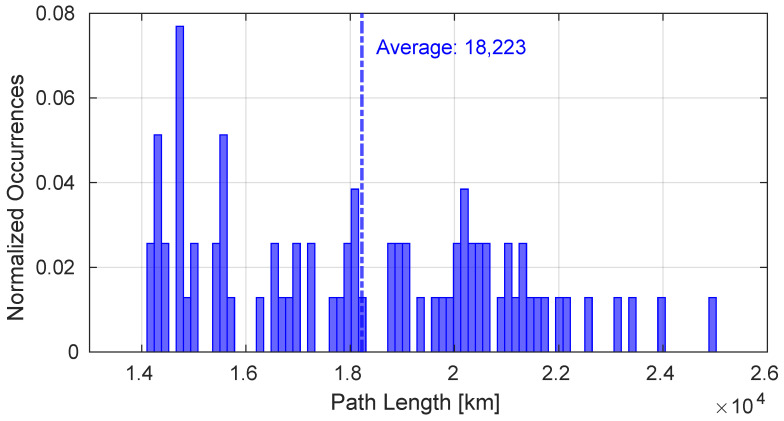
Length of generated E2E paths.

Figure 5 shows the attenuation of G2S links throughout the duration of the entire simulation calculated according to Equation (2) compared to the attenuation values of the inter-satellite links composing the E2E paths. The paths generated during the entire simulation were composed of 6 satellites. Despite the fact that the atmospheric attenuation component is not present in the inter-satellite links, in some cases the attenuation values are superior with respect to those obtained in the G2S links. In fact, as depicted in Figure 6, the inter-satellite links have a greater average distance than the G2S links, and this has a significant impact on attenuation. The total path lengths are represented in Figure 7.

**Figure 8:**
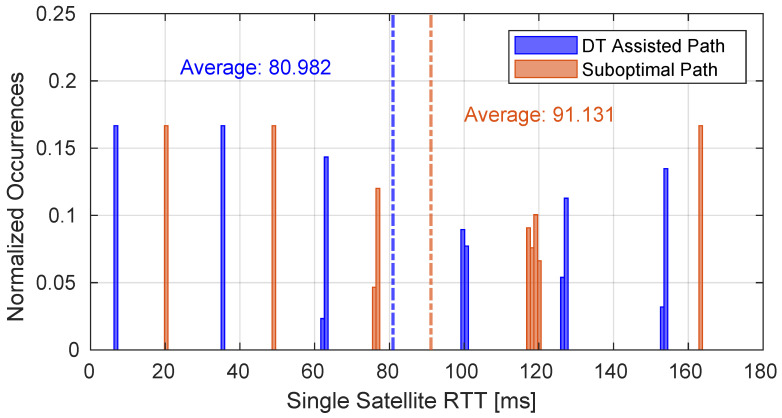
Performance of the RTT reaching a single satellite of the E2E path according to the optimization performed by the DT compared with the performance of a suboptimal one.

In Figure 8, the performance is depicted in terms of the RTT required to reach the single satellites composing the path derived from the processing performed by the DT, compared to the performance of a suboptimal path. As can be observed, in the case where the DT is employed, the performance improves by 10%.

**Figure 9:**
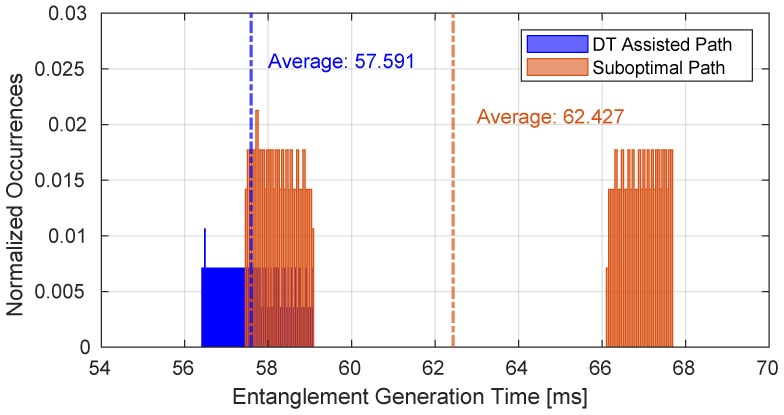
Performance of the best path configured according to the optimization performed by the DT compared with a suboptimal one.

Figure 9 shows the performance of the best path configured according to the optimization performed by the DT compared with a less durable one with respect to a specific time interval. As can be seen, the time required to obtain entanglement is lower in the case of the best path, and the continuity of its distribution indicates that no disconnections occur during the considered time interval. This is due to the fact that the path is connected during the time interval in which the GSs are simultaneously in visibility with their related satellites; after this time interval has elapsed, it is necessary to establish another path that requires some time to reconnect. The connection time is mainly influenced by the pointing time in addition to the setup phase of the protocol. In fact, as can be seen from Figure 9, the histogram is not continuous because the suboptimal path consists of two consecutive paths, both of which exhibit worse performance compared to the DT assisted one whose duration equals the sum of the two suboptimal paths.

**Figure 10:**
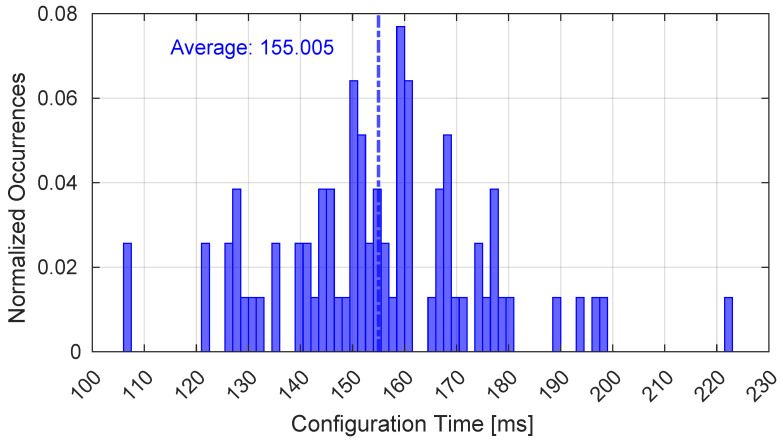
Time interval required for the configuration of the E2E path.

Moreover, Figure 10 shows the time required to configure the E2E path through the loop part of the proposed protocol, represented in Figure 4. It is important to note that only the time required to cross the constellation satellites is considered here and not the time related to the terrestrial path from the MCS to the GSs, which does not depend on the constellation kinematics.

**Figure 11:**
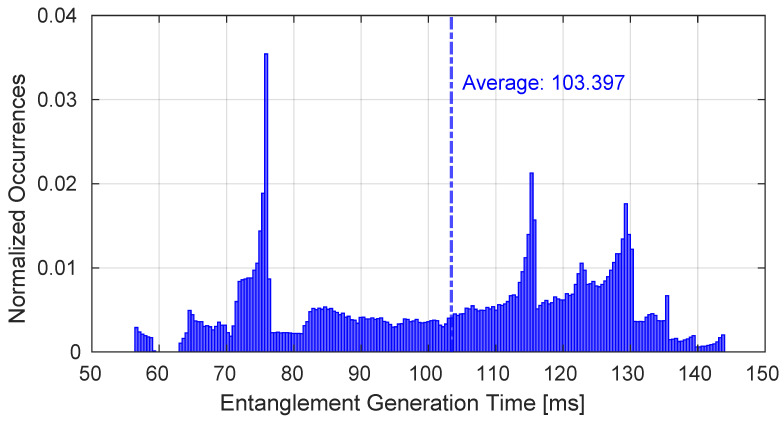
Time required to obtain the entanglement on the paths generated during the entire simulation.

Figure 11 represents the measured latency to obtain an entanglement between the couple of GSs. Considering that although it is a TG, the obtained paths have a lifetime equal to the related reference time interval. Furthermore, as shown from Figure 11, the average latency value is around 100 ms.

**Figure 12:**
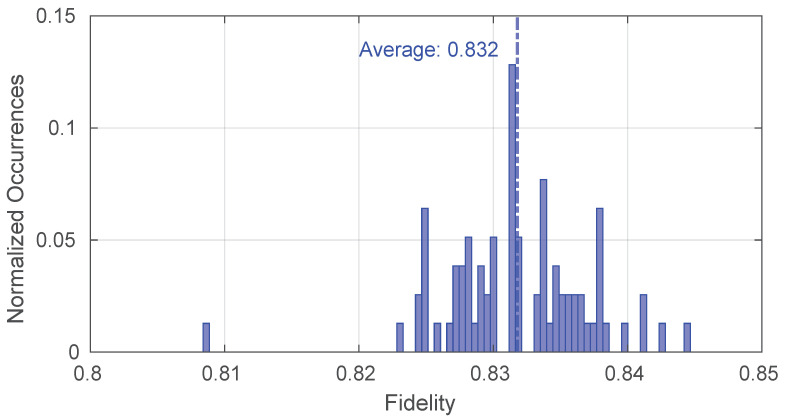
Fidelity values obtainable on the satellite repeater chain.

Furthermore, we evaluated the achievable fidelity on the satellite paths under optimal atmospheric conditions. In particular, Figure 12 illustrates that values above 0.8 can be attained, thereby demonstrating that the quantum states produced have a fidelity that is above the minimum acceptable threshold.

An additional study on the scalability of the entire system could provide significant data for the creation of an efficient architecture where the MCS manages a large number of users by evaluating the capabilities under certain conditions.

## 6. Conclusions

Recently, there have been significant developments in different kinds of QDs, and several experiments employing single satellites have been conducted. Therefore, it is fundamental to deploy a specific QSB based on the principles of quantum entanglement and teleportation with the aim of connecting QCs on Earth to create a distributed quantum system with a computational capacity that goes far beyond the capabilities of any classical distributed computing system. The launch of a specific QSB will allow overcoming the limitations of OF links; therefore, also considering the technological advances in quantum satellite communications, we were motivated to conceive a system for the management of a LEO QSB. Taking into account the difficulties that are encountered in the creation of entanglement over long distances, a DT-assisted SDN QSB could be significant in making the process as efficient as possible, controlling the operations necessary to generate an E2E entanglement. In particular, we have conceived a dedicated architecture comprising a DT and an SDN Controller on the ground that controls the operations to configure the selected E2E paths. In addition, we have defined a dedicated protocol to configure the path based on the results provided by the DT to ensure efficient entanglement distribution on an E2E basis, optimizing, in particular, the monitoring procedure of the involved satellites performed by the DT. Specifically, we analyzed in terms of the attenuation, the links composing the E2E paths generated by the algorithm applied to the TG. Moreover, we evaluated the performance of the system by considering the time required to configure E2E paths, the time required to generate entangled states on an E2E basis, the RTT required to reach the satellites that compose the path, and the fidelity.

The protocol has been evaluated at varying network conditions and thus the E2E paths, which are highly dependent on the variation of the satellite positions with respect to the GSs, thus evaluating the time required to configure the paths selected by the algorithm. From the performed simulations, it is possible to infer that the developed approach is efficient, considering that it guarantees as few as possible disconnections and the time required to reach the involved satellites is lower with respect to a system that does not include an optimization performed by a DT. Furthermore, the average entanglement rate achieved is significant, especially considering the potentially long connection recovery times, which are further impacted by the response time of the pointing mechanisms.

Future development could consist of the research of new DT quantum satellite scenarios with the aim to improve the performance further also considering scenarios with a higher number of users.

## Figures and Tables

**Table 1:** Values of the parameters used to calculate attenuation as in Formula (2). $F{\left(\rho ,\sigma \right)}^{*}$ is the typical fidelity threshold.

Parameters	λ	$D_{R}$	$D_{T}$	$T_{T}$	$T_{R}$	$L_{P}$	$T_{\mathrm{OPT}}$	PDE	$r_{0}$	$F{\left(\rho ,\sigma \right)}^{*}$
**Values**	1550 nm	0.8 m	0.3 m	0.8	0.8	0.2	0.35	0.9	0.1 m	0.8

## Data Availability

Data is contained within the article.
